# Characterization of patients treated at a rare disease referral service: a descriptive study, 2016-2021

**DOI:** 10.1590/S2237-96222024v33e20240204.en

**Published:** 2024-12-20

**Authors:** Everson Andrade de Carvalho, Roberto Del Claro Hopker, Gustavo Henrique Pedroso, Leonardo Silva de Almeida, José Alfredo Trevisan Pacher, André Lucas Miranda Antônio, Josiane de Souza, Michelle Silva Zeny, Mara Lúcia Schmitz Ferreira Santos, Daniel Almeida do Valle, Fabiana Antunes Andrade

**Affiliations:** 1 Universidade Positivo, Departamento de Medicina, Curitiba, PR, Brazil; 2 Hospital Pequeno Príncipe, Ambulatório de Doenças Raras, Curitiba, PR, Brazil

**Keywords:** Rare Diseases, Epidemiology, Tertiary Health Care, Effective Access to Health Services, Diagnosis, Enfermedades Raras, Epidemiología, Atención Terciaria de Salud, Acceso Efectivo a los Servicios de Salud, Diagnóstico

## Abstract

**Objective:**

To analyze the first referral service for rare diseases accredited by the Brazilian Ministry of Health, focusing on referral from the primary healthcare network through to diagnosis.

**Methods:**

This is a descriptive study with patients treated between 2016 and 2021 at a referral hospital service located in Curitiba, Paraná, Brazil. Clinical and epidemiological data were obtained from medical records, as were the results of genetic tests at the hospital’s clinical analysis laboratory. Qualitative data were expressed as absolute and relative frequencies, while quantitative data were expressed as medians and interquartile ranges and compared using the Kruskal-Wallis test.

**Results:**

The study included 1,751 cases, 34.1% were diagnosed with rare diseases, with average time until diagnosis being 3.0 years, whereby mucopolysaccharidosis type II (4.0%) and tuberous sclerosis (3.9%) were the most common. Greater length of time for obtaining diagnosis (p-value 0.004) and receiving specialized care (p-value<0.001) was found in patients from the interior region of Paraná state, compared to those residing in Curitiba city and its metropolitan region.

**Conclusion:**

Diagnosis of rare diseases occurred in approximately one third of cases. The average time until diagnosis suggests a possible positive impact of implementing the referral service. The longer time until diagnosis and specialized care found among patients from the interior region of Paraná represent challenges regarding adequate referral to specialized services.

## Resumen

**Objetivo:**

Analizar el primer servicio de referencia de enfermedades raras acreditado por el Ministerio de Salud, centrándose en la derivación desde la red primaria al diagnóstico.

**Métodos:**

Se trata de un estudio descriptivo con pacientes atendidos entre 2016 y 2021 en un servicio hospitalario de referencia ubicado en Curitiba, Paraná, Brasil. Los datos clínicos y epidemiológicos se obtuvieron de los registros médicos y de los resultados de las pruebas genéticas del laboratorio de análisis clínicos del hospital. Los datos cualitativos se expresaron como frecuencias absolutas y relativas, y los datos cuantitativos como mediana y rango intercuartílico y se compararon mediante la prueba de Kruskal-Wallis.

**Resultados:**

Se incluyeron 1.751 pacientes, el 34,1% fueron diagnosticados con enfermedades raras, con un tiempo promedio de 3,0 años, siendo las más frecuentes la mucopolisacaridosis tipo II (4,0%) y la esclerosis tuberosa (3,9%). Se observó mayor tiempo para obtener el diagnóstico (valor de p-valor 0,004) y atención especializada (valor de p-valor<0,001) en los pacientes del interior de Paraná, en comparación con los residentes en Curitiba y la región metropolitana.

**Conclusión:**

El diagnóstico de enfermedades raras se produjo en aproximadamente un tercio de los casos. El tiempo medio hasta el diagnóstico sugiere un posible impacto positivo de la implementación del servicio de referencia. El mayor tiempo hasta el diagnóstico y la atención especializada observados en pacientes del interior de Paraná representan desafíos en la adecuada derivación a unidades especializadas.

## Introduction

Rare Diseases are a heterogeneous group of conditions, many chronically debilitating, which have a high impact on the quality of life of patients and families ^1^. Rare diseases are those that affect up to 65 people out of every 100,000 individuals, 80% of which are due to genetic factors, placing an important burden on public health ^2^. Around 7000 different rare diseases have been described, having highly diverse signs and symptoms, creating a challenge for diagnosis and therapeutic management of these patients ^3^. It is estimated that between 50% and 70% of rare diseases are pediatric in onset ^4^, with 30% of affected children not surviving beyond five years old, highlighting the importance of adequate management and early diagnosis of these diseases ^5^.

The prevalence of people with rare diseases in Brazil is not completely known ^5^. It is estimated that around 2% to 3% of live newborns have some congenital anomaly, determined at least in part by genetic factors ^6^, responsible for the second leading cause of infant death across the country and for around one third of pediatric hospitalizations ^7^. Furthermore, rare genetic diseases can lead to developmental delay, intellectual impairment, neurodegeneration, among other relevant impacts on the patient’s health ^2^.

Given the relevance of the topic, in 2014 the Ministry of Health established the National Policy for Comprehensive Care for People with Rare Diseases, within the scope of the Brazilian National Health System (Sistema Único de Saúde - SUS), with the aim of expanding access to diagnosis, treatment, prevention and rehabilitation of these patients, with interdisciplinary professional action ^8^. Currently, the Ministry of Health has around 17 specialized establishments qualified to care for rare diseases, distributed in different regions of Brazil ^9^.

Despite several initiatives to address the challenges associated with rare diseases, efforts need to be concentrated ^1^. Among the main challenges faced by patients with rare diseases and their families is limited access to health services, lack of specialized clinical personnel and specific infrastructure, as well as limited access to genetic tests ^8^, which results in delays in diagnosis and inadequate monitoring. The limited number of specific interventions for these diseases also stands out ^1^. Scarce use of resources destined to research on the topic contributes to the delay in scientific and technological development in the area, reflected in the reduced number of scientific publications, as well as the lack of more precise epidemiological studies on rare diseases ^4^.

As such, the objective of this study was to analyze the first Rare Disease Referral Service accredited by the Ministry of Health, focusing on investigating the referral process from the primary healthcare network through to diagnosis.

Methods

### Design and context

This is an observational descriptive study conducted at *Hospital Pequeno Príncipe*, in Curitiba, Paraná, Brazil. In 2016, the first rare disease referral service accredited by the Ministry of Health was opened at this pediatric hospital. In 2024, the hospital had 369 beds, being a national referral center for highly complex pediatric treatment. Around half cases come from Curitiba and its metropolitan region and the remainder from other cities in Paraná and other states.

### Participants

Consecutive patients with rare diseases treated at the Rare Diseases Outpatient Clinic between 2016 and 2021 were included, with no age or sex restrictions.

### Variables

The variables investigated were: sex (female, male), age (years: <1, 1-4, 5-8, 9-12, 13-18, ≥18), race/skin color (White, mixed race, Black, Asian), patient’s place of origin (interior region of Paraná, Curitiba itself, Curitiba’s metropolitan region, the coast, other states, abroad), signs and symptoms present at admission (morphological, behavioral, cognitive changes, developmental delay), diagnosis date, age at first symptoms, date of admission to the referral service, etiology of the rare disease (genetic or non-genetic) and diagnostic resource used (clinical, immunological, metabolic criteria, genetic evaluation). 

### Data source and measurement

Clinical and epidemiological data were collected via retrospective analysis of data taken from the referral service medical records, as well as genetic test results accessible through the interface between the clinical analysis laboratory and the hospital. Genetic test results were classified as positive when the presence of a causal, pathogenic or probably pathogenic genetic mutation was indicated and negative when the absence of evidence of a causal variant or a significant variant was reported. Genetic assessments by means of gene panels, exomes and genomes, were performed using next-generation sequencing.

Diagnosis date was defined as the positive genetic test result date or the date shown on the patient’s medical record. Time until diagnosis was defined as the period between symptom onset and diagnosis. In the case of prenatal diagnoses and pre-symptomatic diagnoses, it was assumed that the diagnosis was made at birth, so that the date of symptom onset and diagnosis coincided, where time until diagnosis was 0. Time until obtaining tertiary care was defined as the period between symptom onset and first care at the hospital under study. 

### Bias control

Data collection was conducted by nine researchers, collaboratively and without blinding. The collection instrument was developed by the researchers themselves in Excel, based on the variables of interest to the research. Before data collection, the researchers underwent training on the correct use of the digital medical record system, as well as the correct interpretation of the medical information held on the medical records. Pilot data collection was done first, covering 10% of the sample, in order to gain experience in doing data collection and potential limitations. The pilot data collected was included in the final sample of the study, as no changes were made to the collection instrument.

### Statistical methods

The Kolmogorov-Smirnov test was applied to assess data normality. Qualitative variables were presented as absolute (n) and relative frequencies (%). Quantitative results were expressed as medians and interquartile ranges, means and minimum and maximum values. Quantitative data were compared using the Kruskal-Wallis test and Dunn’s test as post-hoc analysis. P-values<0.05 were considered significant. The statistical analyses were performed with the aid of SPSS 17.0.

## Results

A total of 1,751 cases were included, of which 56.7% (n=993) were male and 43.3% (n=758) were female. At the first consultation, the majority were 1-4 years old (25.9%), followed by 5-8 years old (20.2%), with an average age of 6.1 years (minimum 0; maximum 34; median 5.0; interquartile range 2.0; 10.0). Most patients were characterized as being of White race/skin color (49.2%), originating mainly from cities in the interior region of the state of Paraná (n=669; 38.2%), the city of Curitiba (n=482; 27.5%) and its metropolitan region (n=375; 21.4%) (Table 1).

**Table 1:** Main general characteristics of patients cared for at a rare disease referral service. Curitiba, Paraná, Brazil, 2016-2021 (n=1,751)

Characteristics	n (%)
**Sex**	
Male	993 (56.7)
Female	758 (43.3)
**Origin**	
Interior region of Paraná	669 (38.2)
Curitiba	482 (27.5)
Metropolitan region	375 (21.4)
Coast	66 (3.8)
Other states	51 (2.9)
Abroad	1 (0.1)
No information	107 (6.1)
**Race/skin color**	
White	862 (49.2)
Mixed race	156 (8.9)
Black	12 (0.7)
Asian	5 (0.3)
No information	716 (40.9)
**Age at first consultation (years)**	
<1	263 (15.0)
1-4	453 (25.9)
5-8	354 (20.2)
9-12	229 (13.1)
13-18	208 (11.9)
≥18	19 (1.1)
No information	225 (12.8)

**Figure 1:**
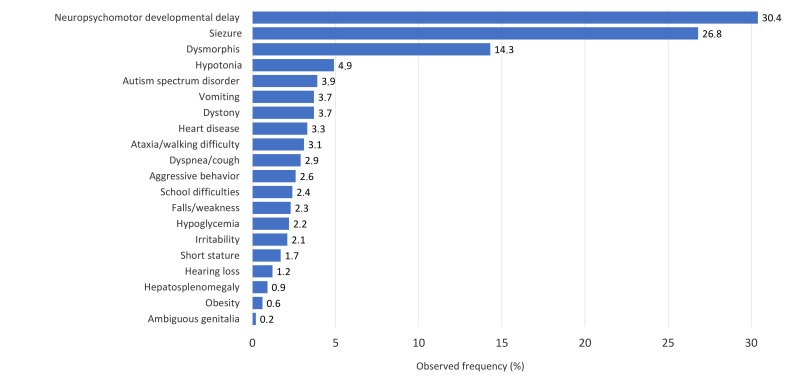
Main signs and symptoms found in patients cared for at a rare disease referral service according to the result obtained. Curitiba, Paraná, Brazil, 2016-2021 (n=1.751)

The most frequent signs and symptoms were neuropsychomotor developmental delay (n=496; 30.4%), followed by seizures (n=437; 26.8%) and dysmorphisms (n=250; 14.3%) (Figure 1), which occurred mainly at age <1 year (20.4%) and between 1-4 years old (19.9%) (mean 2.9; minimum 0; maximum 25; median 1.0; interquartile range 0.2; 4.0 years) (Table 2).

**Table 2:** Symptom onset, diagnosis and specialized care of patients cared for at a rare disease referral service. Curitiba, Paraná, Brazil, 2016-2021 (n=1.751)

Variables	n (%)
**Age at sign and symptom onset (years)**	
Ao nascimento	180 (10.3)
<1	358 (20.4)
1-4	348 (19.9)
5-8	116 (6.6)
9-12	80 (4.6)
13-18	58 (3.3)
≥18	1 (0.1)
No information	610 (34.8)
**Diagnosis obtained**	
Yes	597 (34.1)
No	1154 (65.9)
**Rare disease etiology**	
Genetic	573 (95.9)
Non-genetic	24 (4.1)
**Time until diagnosis (years)**	
<1	81 (13.5)
1-2	77 (12.9)
3-4	36 (6.0)
5-6	15 (2.5)
7-8	15 (2.5)
9-12	11 (1.8)
13-16	7 (1.2)
No information	355 (59.5)
**Time until tertiary care (years)**	
<1	455 (26.0)
1-4	364 (20.8)
5-8	135 (7.7)
9-12	63 (3.6)
13-18	35 (2.0)
≥18	4 (0.2)
No information	695 (39.7)

In all, 597 (34.1%) were diagnosed as having a rare disease, the majority of which had genetic etiology (95.9%), whereby average time until diagnosis was 3.0 years (minimum 0; maximum 16; median 1.8; interquartile range: 0.6; 4.2) and average time until care in a tertiary hospital was 3.1 years (minimum 0; maximum 27; median 1.4 years; interquartile range 0.3; 4.6 ) (Table 2). Among the rare diseases diagnosed, mucopolysaccharidosis type II (4.0%), tuberous sclerosis (3.9%) and anti-N-methyl-D-aspartate receptor encephalitis (1.8%) were the most common. Among the diagnostic methods used, gene panels investigating groups of genes by next-generation sequencing (37.0%), karyotyping (18.5%) and microarray-based comparative genomic hybridization (17.4%) were the most used (Table 3). 

**Table 3:** Methods used in the diagnosis of patients cared for at a rare disease referral service according to the result obtained. Curitiba, Paraná, Brazil, 2016-2021 (n=597)

Diagnosis method	Total	Positive (%)	Negative (%)
Diagnosis using clinical criteria	35 (2.2)	35 (100.0)	0
Immunological	68 (4.3)	14 (20.6)	54 (79.4)
Metabolic	73 (4.6)	71 (97.3)	2 (2.7)
Karyotype	294 (18.5)	15 (5.1)	279 (94.9)
Fluorescence in situ hybridization	1 (0.1)	1 (100.0)	0
Multiplex ligation-dependent probe amplification	11 (0.7)	5 (45.5)	6 (54.5)
Microarray-based comparative genomic hybridization	278 (17.5)	92 (33.1)	186 (66.9)
Gene panels	589 (37.0)	156 (26.5)	433 (73.5)
Exome	181 (11.4)	99 (54.7)	82 (45.3)
Genome	29 (1.8)	20 (69.0)	9 (31.0)
Other	32 (2.0)	11 (34.4)	21 (65.6)

Time until diagnosis for patients from the interior region of the state was 2.3 years, on average, while for those living in Curitiba it was 1.3 years (p-value 0.004) (Table 4). Getting tertiary care was also found to take longer among those living in the interior region of the state when compared those living in Curitiba and its metropolitan region (p-value<0.001) (Table 4).

**Table 4:** Median time (interquartile range) in years until diagnosis obtained (n=242) and tertiary care (n=1,056) according to the origin of patients cared for at a rare disease referral service. Curitiba, Paraná, Brazil, 2016-2021

Origin	Diagnosis	p-value	Tertiary care	p-value
Curitiba	1.3 (0.3; 3.6)		0.4 (0.03; 1.4)	
Metropolitan region	1.3 (0.7; 3.9)	0,004	0.5 (0.2; 1.2)	<0.001
Interior region of Paraná	2.3 (1.2; 5.0)		1.6 (0.4; 5.1)	

## Discussion

Rare diseases represent a significant global public health challenge (10) and also present several specific challenges, among which is the scarcity of robust epidemiological studies that can assist with the definition of public policies and planning of assistance and comprehensive care for these patients (2,3). Our results highlight the complexity involved in diagnosing rare diseases, as diagnosis was only achieved for a third of the cases. Delay in referral, especially for patients living in the interior region of Paraná, may be a factor contributing to average time until diagnosis being below that recommended by specialized international societies, although it is a shorter time than that reported by other international studies on rare diseases, which points to favorable impacts arising from the implementation of the referral service we studied.

Evaluating efficiency in health program implementation requires specific health evaluation research, with its own methodology, which could not be applied in this study, thus comprising one of its limitations. Furthermore, the lack of complete clinical data in the medical records of some patients is also a limitation and may lead to inaccurate interpretations of the results.

In this study, the main reason for referral was the presence of neuropsychomotor developmental delay and epilepsy, which suggests that Rare Diseases may be suspected of occurring in patients with neurological conditions. However, since these signs are not specific to rare or genetic conditions, they continue to be an important factor contributing to delays in diagnosis and referral to specialized care ^11^.

The most common age of care provision, up to 4 years, and the manifestation of the first signs and symptoms before 12 months old, highlight the ages that require greater attention from caregivers. About 50% of rare diseases are diagnosed in early childhood ^12^, in line with the findings of this research. 

In this study, average time between the appearance of the first symptoms and diagnosis was three years. Some international studies indicate an average time until diagnosis of rare diseases that varies between four and six years ^13^ ^-^ ^15^. Average time until diagnosis lower than the general range observed in previous studies may be due to a positive effect resulting from the implementation of the referral service we studied here, despite its short period of operation and the complexity inherent to rare diseases. The long-term search for an accurate diagnosis of rare diseases, referred to as a “diagnostic odyssey,” often leads to high clinical, psychosocial, and economic suffering for patients, as well as their families ^16^. The process leading to diagnosis of rare diseases generally involves visits to different specialists, numerous tests, travelling to specialized centers outside the patient’s region of residence, inadequate treatments, increased severity of complications arising from the genetic condition, high rate of hospitalizations, among other problems, thus emphasizing the importance of early diagnosis ^17^.

Although time until diagnosis was shorter than that identified in other studies, the International Rare Diseases Research Consortium has set the target for all rare diseases to be diagnosed within one year after the first consultation motivated by symptoms ^18^. Time until diagnosis was longer than recommended for more than half the patients in our study, with considerable latency until accessing the referral service. Several factors contribute to this delay in obtaining diagnosis, including high clinical heterogeneity, non-specific symptoms, variable course of the disease among patients and delays in seeking and referring to specialized services ^19^. Following hospital admission, the average time until diagnosis was relatively short, considering the complexity of the conditions involved. This data highlights the importance of strengthening the primary health network to recognize warning signs and suspected cases of rare diseases ^16^, thus enabling early referral to specialized services and consequent early diagnosis.

The majority of patients treated at the referral service lived in cities in the interior region of Paraná. These patients faced greater delays in diagnosis, possibly due to difficulty in accessing specialized care, since time until tertiary care was also longer in this group of patients. These findings highlight the importance of professionals working in primary health care recognizing patients who need to be referred to a referral service ^8^, in addition to providing guidance on primary prevention measures that can reduce the risk of congenital anomalies ^16^. 

The longer length of time until diagnosis of rare diseases observed among children living in the interior region of Paraná, compared to those in the state capital, reflects significant challenges in both recognizing them and appropriate referral to specialized services ^16^. Difficulty in accessing specialized services in the interior region contributes to this delay. Centralization of rare disease services in the state capital is a factor that makes the diagnostic process difficult for the population that lives in the interior region ^9^. Delays may also be related to access to health services via the SUS nationwide. Due to the specificity of rare diseases, access difficulties can be even more pronounced ^5^.

Obtaining accurate diagnoses for rare diseases often depends on access to molecular diagnostic tests, which are not yet widely available ^9^. The next-generation sequencing technique has potentially accelerated the pace of rare disease diagnoses compared to standard genetic testing approaches ^20^ ^-^ ^22^. High frequency of use of karyotype testing was found in our study, it being a lower cost and lower resolution test, generally requested as initial screening for major chromosomal mutations ^23^. Investigation using gene panels was the most used method, with higher positivity, although Next-Generation Sequencing applied to the assessment of whole exome and genome sequencing showed higher rates, which is in line with the literature ^24^ ^,^ ^25^. Use of Next-Generation Sequencing may have contributed to the shorter average time until diagnosis found in our study. However, it is important to note that whole exome and genome sequencing analyses are expensive and, in many cases, are not covered by health care insurance ^26^. Pharmacoeconomic analyses of whole exome and genome sequencing, considering the cost of the tests and their effectiveness and comparing them with scaling techniques, would be necessary in order to improve health policy in the area.

In conclusion, neuropsychomotor developmental delay and seizures in early childhood were relevant signs of suspected rare diseases in a referral service in Curitiba. Average time until diagnosis suggests a positive impact of implementing the referral service we studied, and delay in diagnosis and referral of patients living in the interior region of the state of Paraná are critical aspects that highlight the need to improve access to referral services. The findings emphasize the need to expand specialized services within the SUS, continuing education for primary health care professionals and training for existing referral services.

## Data Availability

Data generated during the study are available from the corresponding author upon request.
