# Supplementary material for: Characterization of patients treated at a rare disease referral service: a descriptive study, 2016-2021
Source: Epidemiol Serv Saude. 2024 Dec 20;33:e20240204. doi: 10.1590/S2237-96222024v33e20240204.en (PMC11660309; doi:10.1590/S2237-96222024v33e20240204.en)
Supplement: Translation

# Translation: Caracterização de pacientes atendidos em serviço de referência em doenças raras: estudo descritivo, 2016-2021

**DOI:** 10.1590/S2237-96222024v33e20240204.pt

**Authors:** Everson Andrade de Carvalho, Roberto Del Claro Hopker, Gustavo Henrique Pedroso, Leonardo Silva de Almeida, José Alfredo Trevisan Pacher, André Lucas Miranda Antônio, Josiane de Souza, Michelle Silva Zeny, Mara Lúcia Schmitz Ferreira Santos, Daniel Almeida do Valle, Fabiana Antunes Andrade

**Affiliations:** 1 Universidade Positivo, Departamento de Medicina, Curitiba, PR, Brasil; 2 Hospital Pequeno Príncipe, Ambulatório de Doenças Raras, Curitiba, PR, Brasil

**Keywords:** Doenças Raras, Epidemiologia, Atenção Terciária à Saúde, Acesso Efetivo aos Serviços de Saúde, Diagnóstico

## Abstract

**Objetivo:**

Analisar o primeiro serviço de referência em doenças raras credenciado pelo Ministério da Saúde, com foco no encaminhamento desde a rede primária até o diagnóstico.

**Métodos:**

Trata-se de um estudo descritivo com pacientes atendimentos entre 2016 e 2021 em serviço hospitalar de referência localizado de Curitiba. Dados clínicos e epidemiológicos foram obtidos de registros médicos e os resultados de exames genéticos do laboratório de análises clínicas do hospital. Dados qualitativos foram expressos como frequência absoluta e relativa, e os quantitativos por mediana e intervalo interquartil e comparados pelo teste Kruskal-Wallis.

**Resultados:**

Foram incluídos 1.751 pacientes, 34,1% obtiveram diagnóstico de doenças raras, com tempo médio de 3,0 anos, sendo a mucopolissacaridose tipo II (4,0%) e esclerose tuberal (3,9%) as mais frequentes. Maior tempo até obter diagnóstico (p-valor 0,004) e atendimento especializado (p-valor<0,001) foram observados em pacientes do interior do Paraná, em comparação com aqueles que residem em Curitiba e região metropolitana.

**Conclusão:**

O diagnóstico de doenças raras ocorreu em cerca de um terço dos casos. O tempo médio até o diagnóstico sugere possível impacto positivo da implementação do serviço de referência. Maior tempo até o diagnóstico e atendimento especializado observado em pacientes do interior do Paraná representam desafios no encaminhamento adequado para unidades especializadas.

## Introdução

As Doenças Raras são um grupo heterogêneo de condições, muitas cronicamente debilitantes, que apresentam elevado impacto na qualidade de vida do paciente e dos familiares ^1^. Doenças raras são aquelas que afetam até 65 pessoas em cada 100 mil indivíduos, sendo que 80% são decorrentes de fatores genéticos, conferindo importante carga à saúde pública ^2^. Já foram descritas cerca de 7 mil diferentes doenças raras, as quais apresentam elevada diversidade de sinais e sintomas, conferindo um desafio para o diagnóstico e o manejo terapêutico desses pacientes ^3^. Estima-se que entre 50% e 70% das doenças raras são de início pediátrico ^4^, sendo que 30% das crianças afetadas não sobrevivem mais que cinco anos de vida, reforçando a importância do manejo adequado e diagnóstico precoce dessas doenças ^5^.

A prevalência de pessoas com doenças raras no território nacional não é completamente conhecida ^5^. Estima-se que em torno de 2% a 3% dos recém-nascidos vivos apresentem alguma anomalia congênita, determinada pelo menos em parte por fatores genéticos ^6^, responsável pela segunda maior causa de morte infantil em todo o país e por cerca de um terço dos internamentos pediátricos ^7^. Além disso, doenças genéticas raras podem levar a atraso no desenvolvimento, deficiência intelectual, neurodegeneração, entre outros relevantes impactos na saúde do paciente ^2^.

Diante da relevância do tema, em 2014 o Ministério da Saúde estabeleceu a Política Nacional de Atenção Integral às Pessoas com Doenças Raras, no âmbito do Sistema Único de Saúde (SUS), com o objetivo de ampliar o acesso ao diagnóstico, ao tratamento, à prevenção e à reabilitação desses pacientes, com atuação profissional interdisciplinar ^8^. Atualmente, o Ministério da Saúde conta com cerca de 17 estabelecimentos especializados e habilitados para o atendimento em doenças raras, distribuídos em diversas regiões do Brasil ^9^.

Apesar de várias iniciativas para enfrentar os desafios associados às doenças raras, esforço precisa ser concentrado ^1^. Entre os principais desafios enfrentados pelos pacientes com doenças raras e seus familiares está o limitado acesso a serviços de saúde, falta de pessoal clínico especializado e infraestrutura específica, bem como limitado acesso a testes genéticos ^8^, o que repercute em atraso no diagnóstico e acompanhamento não adequado, além disso, destaca-se o restrito número de intervenções específicas para essas doenças ^1^. O escasso emprego de recursos destinados à pesquisa do tema contribui para o atraso do desenvolvimento científico e tecnológico na área, refletindo no reduzido número de publicações científicas, bem como na falta de estudos epidemiológicos mais precisos sobre doenças raras ^4^.

Assim, o presente estudo tem como objetivo analisar o primeiro Serviço de Referência em Doenças Raras credenciado pelo Ministério da Saúde, com foco na investigação do processo de encaminhamento desde a rede primária até o diagnóstico.

## Métodos

### Delineamento e contexto

Trata-se de um estudo observacional do tipo descritivo realizado no Hospital Pequeno Príncipe, em Curitiba, Paraná. Em 2016, foi habilitado o primeiro serviço de referência de doenças raras credenciado pelo Ministério da Saúde neste hospital pediátrico. Em 2024, o hospital contava com 369 leitos, sendo centro de referência nacional para tratamentos de alta complexidade em pediatria. Cerca de metade dos atendimentos são provenientes de Curitiba e região metropolitana e demais, de outras cidades do Paraná e de outros estados.

### Participantes

Foram incluídos pacientes consecutivos com doenças raras atendidos no Ambulatório de doenças raras no período de 2016 e 2021, sem restrição de faixa etária ou sexo. 

### Variáveis

As variáveis investigadas foram: sexo (feminino, masculino), idade (anos: <1, 1-4, 5-8, 9-12, 13-18, ≥18), raça/cor da pele (branca, parda, preta, amarela), local de origem do paciente (interior do Paraná, Curitiba, região metropolitana, litoral, outros estados, exterior), sinais e sintomas presentes na admissão (alterações morfológicas, comportamentais, cognitivas, atraso no desenvolvimento), data do diagnóstico, idade dos primeiros sintomas, data da admissão no serviço de referência, etiologia da doença rara (genética ou não genética) e recurso diagnóstico empregado (critérios clínicos, imunológicos, metabólico, avaliação genética). 

### Fonte de dados e mensuração

A coleta de dados clínicos e epidemiológicos se deu via análise retrospectiva de dados de prontuários do serviço de referência, bem como dos exames genéticos acessíveis através da interface do laboratório de análises clínicas e o hospital. Os resultados de testes genéticos foram classificados como positivo quando foi indicada a presença de alteração genética causal, patogênica ou provavelmente patogênica e negativo quando foi relatada a ausência de evidência de variante causal ou de variante de significado inserto. As avaliações genéticas através de painéis gênicos, exoma e genoma, foram realizadas por sequenciamento de nova geração.

A data de diagnóstico foi definida como a presente no resultado positivo de exame genético ou aquela descrita no prontuário do paciente. O tempo até o diagnóstico foi definido como o período entre o início dos sintomas e o diagnóstico. No caso dos diagnósticos pré-natais e diagnósticos pré-sintomáticos, assumiu-se que o diagnóstico foi feito ao nascimento, de modo que a data de início dos sintomas e o diagnóstico coincidiram, com tempo até o diagnóstico foi 0. O tempo até obter atendimento terciário foi definido como período entre o início dos sintomas e o primeiro atendimento no hospital de estudo. 

### Controle de viés

A coleta de dados foi conduzida por nove pesquisadores, de forma colaborativa e sem cegamento entre eles. O instrumento de coleta foi desenvolvido pelos próprios pesquisadores no Excel, com base nas variáveis de interesse da pesquisa. Antes da coleta de dados os pesquisadores passaram por treinamento para o correto uso do sistema de prontuários digital, bem como da correta interpretação dos registros médicos apresentados no prontuário. Uma coleta piloto, contemplando 10% da amostra, foi realizada, de forma a vivenciar a prática da coleta de dados e potenciais limitações. A coleta piloto foi incluída na amostra final do estudo, visto que não houve modificação do instrumento de coleta.

### Métodos estatísticos

O teste de Kolmogorov-Smirnov foi aplicado para avaliação da normalidade do dado. As variáveis qualitativas foram apresentadas como frequência absoluta (n) e relativa (%). Os resultados quantitativos foram expressos por medianas e intervalo interquartil, média e valores mínimo e máximo. Os dados quantitativos foram comparados com teste Kruskal-Wallis e teste de Dunn como análise post-hoc e p-valor<0,05 foi considerado significativo. As análises estatísticas foram realizadas com o auxílio do programa SPSS 17.0.

## Resultados

Foram incluídos 1.751, sendo 56,7% (n=993) do sexo masculino e 43,3% (n=758), do feminino. No primeiro atendimento, a maioria tinha 1-4 anos (25,9%), seguida por 5-8 anos (20,2%), com 6,1 anos de idade em média no atendimento (mínimo 0; máximo 34; mediana 5,0; intervalo interquartil 2,0; 10,0). A maior parte dos pacientes foram caracterizados com raça/cor da pele branca (49,2%), com origem, principalmente de cidades do interior do Paraná (n=669; 38,2%), Curitiba (n=482; 27,5%) e região Metropolitana (n=375; 21,4%) (Tabela 1).

**Tabela 1:** Principais características gerais dos pacientes atendidos em serviço de referência de doenças raras. Curitiba, Paraná, 2016-2021 (n=1.751)

Características	n (%)
**Sexo**	
Feminino	993 (56,7)
Masculino	758 (43,3)
**Procedência**	
Interior do Paraná	669 (38,2)
Curitiba	482 (27,5)
Região metropolitana	375 (21,4)
Litoral	66 (3,8)
Outros estados	51 (2,9)
Exterior	1 (0,1)
Sem informação	107 (6,1)
**Raça/cor da pele**	
Branca	862 (49,2)
Parda	156 (8,9)
Preta	12 (0,7)
Amarela	5 (0,3)
Sem informação	716 (40,9)
**Idade na primeira consulta (anos)**	
<1	263 (15,0)
1-4	453 (25,9)
5-8	354 (20,2)
9-12	229 (13,1)
13-18	208 (11,9)
≥18	19 (1,1)
Sem informação	225 (12,8)

Os sinais e sintomas mais frequentes foram o atraso no desenvolvimento neuropsicomotor (n=496; 30,4%), seguido por convulsões (n=437; 26,8%) e dismorfismos (n=250; 14,3%) (Figura 1), os quais ocorreram principalmente com idade <1 ano (20,4%) e entre 1-4 anos (19,9%) (média 2,9; mínimo 0; máximo 25; mediana 1,0; intervalo interquartil 0,2; 4,0 anos). (Tabela 2).

**Figura 1:** Principais sinais e sintomas presentes nos pacientes atendidos em serviço de referência de doenças raras de acordo com o resultado obtido. Curitiba, Paraná, 2016-2021 (n=1.751)

**Tabela 2:** Início dos sintomas, diagnóstico e atendimento especializado de pacientes atendidos em serviço de referência de doenças raras. Curitiba, Paraná, 2016-2021 (n=1.751)

Variáveis	n (%)
**Idade no início dos sinais e sintomas (anos)**	
Ao nascimento	180 (10,3)
<1	358 (20,4)
1-4	348 (19,9)
5-8	116 (6,6)
9-12	80 (4,6)
13-18	58 (3,3)
≥18	1 (0,1)
Sem informação	610 (34,8)
**Diagnóstico obtido**	
Sim	597 (34,1)
Não	1154 (65,9)
**Etiologia da doença rara**	
Genética	573 (95,9)
Não genética	24 (4,1)
**Tempo até diagnóstico (anos)**	
<1	81 (13,5)
1-2	77 (12,9)
3-4	36 (6,0)
5-6	15 (2,5)
7-8	15 (2,5)
9-12	11 (1,8)
13-16	7 (1,2)
Sem informação	355 (59,5)
**Tempo até atendimento terciário (anos)**	
<1	455 (26,0)
1-4	364 (20,8)
5-8	135 (7,7)
9-12	63 (3,6)
13-18	35 (2,0)
≥18	4 (0,2)
Sem informação	695 (39,7)

Um total de 597 (34,1%) obtiveram diagnóstico de doença rara, a maioria das de etiologia genética (95,9%), com média de tempo até o diagnóstico de 3,0 anos (mínimo 0; máximo16; mediana 1,8; intervalo interquartil: 0,6; 4,2) e média de tempo até o atendimento em hospital terciário de 3,1 anos (mínimo 0; máximo 27; mediana 1,4 anos; intervalo interquartil 0,3; 4,6) (Tabela 2). Entre as doenças raras diagnosticadas, a mucopolissacaridose tipo II (4,0%), esclerose tuberal (3,9%) e encefalite antirreceptor de N-metil-D-aspartato (1,8%) foram as mais frequentes. Entre os métodos diagnósticos empregados, os painéis gênicos investigando grupos de genes por sequenciamento de nova geração (37,0%), o cariótipo (18,5%) e hibridização genômica comparativa por microarranjo (17,4%) foram os mais empregados (Tabela 3). 

**Tabela 3:** Métodos empregados no diagnóstico dos pacientes atendidos em serviço de referência de doenças raras de acordo com o resultado obtido. Curitiba, Paraná, 2016-2021 (n=597)

Método diagnóstico	Total	Positivo (%)	Negativo (%)
Diagnóstico por critérios clínicos	35 (2,2)	35 (100,0)	0
Imunológico	68 (4,3)	14 (20,6)	54 (79,4)
Metabólico	73 (4,6)	71 (97,3)	2 (2,7)
Cariótipo	294 (18,5)	15 (5,1)	279 (94,9)
Hibridização in situ por fluorescência	1 (0,1)	1 (100,0)	0
Amplificação multiplex de sondas dependente de ligação	11 (0,7)	5 (45,5)	6 (54,5)
Hibridização genômica comparativa por microarranjo	278 (17,5)	92 (33,1)	186 (66,9)
Painéis gênicos	589 (37,0)	156 (26,5)	433 (73,5)
Exoma	181 (11,4)	99 (54,7)	82 (45,3)
Genoma	29 (1,8)	20 (69,0)	9 (31,0)
Outros	32 (2,0)	11 (34,4)	21 (65,6)

Pacientes do interior do estado apresentaram o tempo até o diagnóstico de 2,3 anos, em média e os residentes em Curitiba foi 1,3 ano (p-valor=0,004) (Tabela 4). Maior tempo até conseguir atendimento terciário também foi observado entre residentes do interior do estado quando comparados aos residentes em Curitiba e região (p-valor<0,001) (Tabela 4).

**Tabela 4:** Mediana (intervalo interquartil) do tempo em anos até obtenção de diagnóstico (n=242) e atendimento terciário (n=1.056) de acordo com a procedência dos pacientes atendidos em serviço de referência de doenças raras. Curitiba, 2016-2021

Procedência	Diagnóstico	p-valor	Atendimento terciário	p-valor
Curitiba	1,3 (0,3; 3,6)		0,4 (0,03; 1,4)	
Região metropolitana	1,3 (0,7; 3,9)	0,004	0,5 (0,2; 1,2)	<0,001
Interior do Paraná	2,3 (1,2; 5,0)		1,6 (0,4; 5,1)	

## Discussão

As doenças raras representam um significativo desafio de saúde pública global ^10^ e apresentam diversos desafios, entre os quais está a escassez de estudos epidemiológicos robustos que possam colaborar na definição de políticas públicas e no planejamento da assistência e cuidado integral desses pacientes ^2^ ^,^ ^3^. Nossos resultados evidenciam a complexidade no diagnóstico das doenças raras, uma vez que ele foi alcançado em apenas um terço dos casos. A demora no encaminhamento, especialmente dos pacientes residentes no interior do Paraná, pode ser um fator contribuinte para o tempo médio até o diagnóstico estar aquém do preconizado pelas sociedades internacionais especializadas, embora seja um tempo inferior ao de outros estudos internacionais sobre doenças raras, o que aponta para impactos favoráveis provenientes da implementação do serviço de referência investigado.

A avaliação da eficiência na implementação de um programa de saúde exige uma pesquisa específica de avaliação em saúde, com metodologia própria, que não pôde ser aplicada neste estudo, constituindo assim uma de suas limitações. Além disso, a ausência de dados clínicos completos nos prontuários de alguns pacientes também representa uma limitação, podendo gerar interpretações imprecisas dos resultados.

No presente estudo, o principal motivo de encaminhamento foi a presença de atraso no desenvolvimento neuropsicomotor e epilepsia, o que sugere a suspeição de Doenças Raras em pacientes com condições neurológicas. No entanto, uma vez que esses sinais não são específicos de condições raras ou genéticas, eles permanecem como um importante fator que contribui para o atraso no diagnóstico e encaminhamento para atendimento especializado ^11^.

A idade mais comum de atendimento, até 4 anos, e a manifestação dos primeiros sinais e sintomas antes dos 12 meses destacam as idades que requerem maior atenção dos cuidadores. Cerca de metade 50% das doenças raras são diagnosticadas primeira infância ^12^, em linha com os achados desta pesquisa. 

No presente estudo, o tempo médio entre o aparecimento dos primeiros sintomas e o diagnóstico foi três anos. Alguns estudos internacionais indicam um tempo médio para diagnóstico de doenças raras que varia entre quatro e seis anos ^13^ ^-^ ^15^. Tempo médio até o diagnóstico inferior à faixa geral observado em estudos prévios pode decorrer de efeito positivo decorrente da implantação do serviço de referência do presente estudo, apesar do curto período de funcionamento e da complexidade inerente às doenças raras. A busca de longo prazo por um diagnóstico preciso para as doenças raras, referida como “odisseia diagnóstica”, geralmente acarreta elevado sofrimento clínico, psicossocial e econômico aos pacientes, bem como para suas famílias ^16^. O processo até o diagnóstico das doenças raras geralmente envolve visitas a diferentes especialistas, numerosos exames, deslocamento para centros especializados fora da região de residência, tratamentos inadequados, elevação da gravidade das intercorrências provenientes da condição genética, elevada taxa de hospitalizações, entre outros problemas, reforçando a importância do diagnóstico precoce ^17^.

Apesar de te sido observado tempo até o diagnóstico inferior ao identificado em outros estudos, o Consórcio Internacional de Investigação em Doenças Raras estabelece como meta que todas as doenças raras sejam diagnosticadas até um ano a após a primeira consulta motivada pelos sintomas ^18^. Mais da metade dos pacientes da presente pesquisa apresentaram tempo superior ao preconizado, com uma latência considerável até a entrada no serviço de referência. Diversos fatores contribuem para essa morosidade na obtenção do diagnóstico, incluindo a elevada heterogeneidade clínica, sintomas não específicos, curso variável da doença entre os pacientes e o atraso na busca e encaminhamento para serviços especializados ^19^. Após a admissão hospitalar, o tempo médio para o diagnóstico foi relativamente curto, considerando a complexidade dos quadros envolvidos. Esse dado destaca a importância do fortalecimento da rede básica de saúde para o reconhecimento de sinais de alerta e suspeição de doenças raras ^16^, possibilitando assim encaminhamento antecipado a serviços especializados e consequente diagnóstico precoce.

A maioria dos pacientes atendidos no serviço de referência residia em cidades do interior do Paraná. Tais pacientes enfrentaram maior atraso diagnóstico, possivelmente devido à dificuldade de acesso a atendimento especializado, já que o tempo até o atendimento terciário também foi superior nesse grupo de pacientes. Esses achados destacam a importância dos profissionais que atuam na atenção básica à saúde no reconhecimento de pacientes que necessitam de encaminhamento para serviço de referência de ^8^, além da orientação sobre medidas de prevenção primária que podem reduzir o risco de anomalias congênitas ^16^. 

O maior tempo de diagnóstico de doenças raras observado entre crianças residentes no interior do Paraná, em comparação com aquelas na capital, reflete desafios significativos tanto no reconhecimento quanto no encaminhamento adequado para unidades especializadas ^16^. A dificuldade de acesso aos centros especializados no interior contribui para esse atraso. A centralização dos centros de doenças raras na capital representa fator que dificulta o processo diagnóstico para a população do interior ^9^. O atraso pode se relacionar também ao acesso aos serviços de saúde pelo SUS em âmbito nacional. Devido à especificidade das doenças raras, as dificuldades de acesso podem ser ainda mais acentuadas ^5^.

A obtenção de diagnósticos precisos em doenças raras geralmente depende do acesso a testes diagnósticos moleculares, os quais ainda não estão amplamente disponíveis ^9^. A técnica de sequenciamento de nova geração potencialmente acelerou o ritmo dos diagnósticos de doenças raras em comparação com abordagens padrão de testes genéticos ^20^ ^-^ ^22^. Elevada frequência de emprego do teste cariótipo foi observada no presente estudo, exame de menor custo e resolução, geralmente solicitado como triagem inicial para alterações cromossômicas maiores ^23^. A investigação por meio de painéis gênicos foi o método mais empregado, com positividade maior, embora o Sequenciamento de Nova Geração aplicado à avaliação do sequenciamento completo de exoma e do genoma tenha apresentado taxas superiores, o que está em conformidade com a literatura ^24^ ^,^ ^25^. O emprego do Sequenciamento de Nova Geração pode ter contribuído para uma menor média de tempo até o diagnóstico observada no presente estudo. No entanto, é importante notar que as análises de Sequenciamento completo de exoma e genoma têm um custo elevado e, em muitos casos, não são cobertas por planos de assistência à saúde ^26^. Análises de farmacoeconomia do sequenciamento completo de exoma e genoma, considerando o custo dos exames e sua efetividade e comparando-os com técnicas escalonadas de investigação, seriam necessárias para o aprimoramento da política de saúde na área.

Em conclusão, o atraso no desenvolvimento neuropsicomotor e convulsões na primeira infância foram sinais relevantes na suspeição de doenças raras em um serviço de referencia de Curitiba. O tempo médio até o diagnóstico sugere um impacto positivo da implementação do serviço de referência estudado, e o atraso no diagnóstico e encaminhamento de pacientes residentes no interior são aspectos críticos que ressaltam necessidade de melhorar o acesso a serviços de referência. Os achados reforçam a necessidade de ampliação de centros especializados no âmbito do SUS, educação continuada para profissionais da atenção primária básica à saúde e treinamento de centros estabelecidos.

## Data Availability

Os dados gerados durante o estudo estão disponíveis com o autor correspondente, mediante pedido
